# Protective Effect of Mesenchymal Stem Cell Active Factor Combined with *Alhagi maurorum* Extract on Ulcerative Colitis and the Underlying Mechanism

**DOI:** 10.3390/ijms25073653

**Published:** 2024-03-25

**Authors:** Xuanhong Cao, Aili Aierken, Jie Wang, Xinrui Guo, Sha Peng, Yaping Jin

**Affiliations:** College of Veterinary Medicine, Northwest A&F University, Yangling, Xianyang 712100, China; stylecxh@163.com (X.C.); ali198908@163.com (A.A.); 17854291993@163.com (J.W.); 2021055522@nwafu.edu.cn (X.G.)

**Keywords:** *Alhagi maurorum*, mesenchymal stem cells, inflammation, ulcerative colitis

## Abstract

Ulcerative colitis (UC) is a relapsing and reoccurring inflammatory bowel disease. The treatment effect of *Alhagi maurorum* and stem cell extracts on UC remains unclear. The aim of the present study was to investigate the protective role of *Alhagi maurorum* combined with stem cell extract on the intestinal mucosal barrier in an intestinal inflammation mouse model. Sixty mice were randomly divided into a control group, model group, Alhagi group, MSC group, and MSC/Alhagi group. MSC and Alhagi extract were found to reduce the disease activity index (DAI) scores in mice with colitis, alleviate weight loss, improve intestinal inflammation in mice (*p* < 0.05), preserve the integrity of the ileal wall and increase the number of goblet cells and mucin in colon tissues. Little inflammatory cell infiltration was observed in the Alhagi, MSC, or MSC/Alhagi groups, and the degree of inflammation was significantly alleviated compared with that in the model group. The distribution of PCNA and TNF-alpha in the colonic tissues of the model group was more disperse than that in the normal group (*p* < 0.05), and the fluorescence intensity was lower. After MSC/Alhagi intervention, PCNA and TNF-alpha were distributed along the cellular membrane in the MSC/Alhagi group (*p* < 0.05). Compared with that in the normal control group, the intensity was slightly reduced, but it was still stronger than that in the model group. In conclusion, MSC/Alhagi can alleviate inflammatory reactions in mouse colonic tissue, possibly by strengthening the protective effect of the intestinal mucosal barrier.

## 1. Introduction

UC is an idiopathic, non-infectious inflammatory disease that has been increasingly prevalent in recent years due to improper lifestyle habits. The global incidence of UC is continuously rising, primarily affecting young individuals. The clinical manifestations of inflammation in patients include rectal bleeding, diarrhea, mucous discharge, abdominal pain, urgency to defecate, fatigue, fever, and weight loss. Although the exact etiology remains unclear, it is widely believed that genetic predisposition, environmental triggers, and abnormal immune response factors contribute to its development. UC typically leads to epithelial cell ulceration and necrosis in the colon [1,2]. Excessive and inappropriate inflammatory reactions along with the presence of certain cytokines (such as IL-1β, IL-8, IL-6) and autoimmune diseases play a central role in colonic inflammation. Research reports suggest that individuals with ulcerative colitis are prone to developing colorectal cancer, osteoporosis, venous thromboembolism, cardiovascular diseases, and other complications that impose a burden on the global economy and healthcare systems [3,4]. Currently, due to an incomplete understanding of its pathogenesis, further research on ulcerative colitis is necessary. There is still significant controversy regarding the differential effectiveness of treatment medications.

Mesenchymal stem cells (MSCs) are derived from the early mesoderm and are pluripotent stem cells that have attracted increasing amount of attention due to their multidirectional differentiation potential and their ability to promote angiogenesis and regulate immune processes, as well as their self-renewal abilities. Moreover, MSCs can secrete related growth factors, including insulin-like growth factor, keratinocyte growth factor, vascular endothelial growth factor, and hepatocyte growth factor [5,6]. It was found that mesenchymal stem-derived cell secretions can inhibit tissue fibrosis, promote angiogenesis, and participate in tissue repair and regeneration [7]. MSC extract is a versatile type of nanoscale particle that possesses characteristics such as stability, tolerance, and the ability to transfer substances, leading it to play an important role in signal transmission [8]. As carriers outside of cells, extracellular vesicles can transmit genetic material and proteins as signals between cells, ultimately providing regulatory functions for many cellular processes including inflammation, angiogenesis, programmed cell death (apoptosis), and scar formation. However, the effect of extracellular vesicles derived from MSC on intestinal fibrosis is yet to be thoroughly studied.

*Alhagi maurorum*, a semi-shrub of the Fabaceae family, is a perennial herbaceous plant. Research on the chemical composition of *Alhagi maurorum* to date has mainly focused on its above-ground parts, revealing that its components are primarily flavonoids, polysaccharides, alkaloids, amino acids, sterols, and other compounds. Flavonoids derived from quercetin and kaempferol are the main constituents [9,10]. The secretion formed by the coagulation of *Alhagi maurorum* leaf exudate is also known as ‘spine sugar’, which contains flavonoid compounds. Although *Alhagi maurorum* has not yet been applied in clinical treatment for sepsis or ulcerative colitis, modern pharmacological studies have shown that extracts from *Alhagi maurorum* have immunomodulatory, anti-allergic, and anti-inflammatory effects with low toxicity [11]. They also exhibit inhibitory effects against Staphylococcus aureus and Escherichia coli; moreover, total flavonoid extracts show significant inhibition against Staphylococcus aureus [12]. These findings suggest that active ingredients in *Alhagi maurorum* may have potential therapeutic effects on UC; however, there is still limited research on the mechanism of action that occurs when using *Alhagi maurorum* to treat UC [13,14]. Network pharmacology analyzes drug–body interactions from a holistic perspective through network analysis of biological systems to elucidate the molecular correlations and patterns between drugs and disease treatments [15]. Traditional Chinese medicine formulas with multiple components targeting multiple pathways align well with the research methods of network pharmacology and can be used to explore discoveries related to active compounds in traditional Chinese medicine, as well as their mechanisms of biological action. Currently, there are no reports on network pharmacology studies investigating the use of *Alhagi maurorum* for treating ulcerative colitis; furthermore, research on *Alhagi maurorum* mainly focuses on pharmacodynamics. Thus, the present study focused on the therapeutic effect of mesenchymal stem cells and *Alhagi maurorum* extracts on dextran–sulfate–sodium-induced UC model mice and the relationship between these effects and the gut microbiota.

## 2. Results

**Figure 1:**
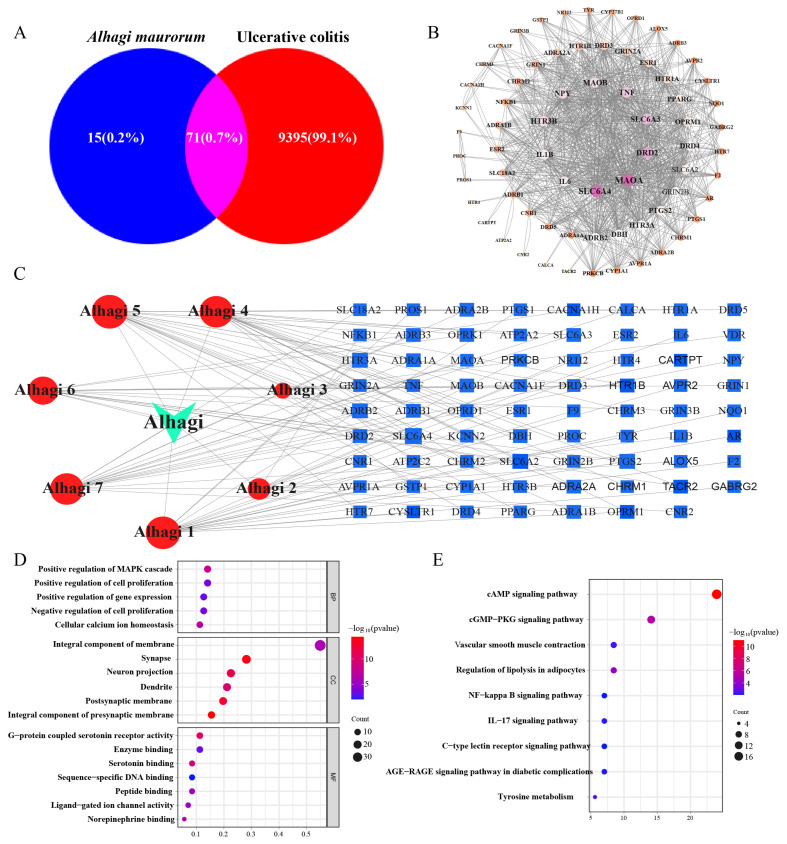
Exploration of the role of *Alhagi maurorum* in ulcerative colitis based on network pharmacology. (**A**) Venn diagram of *Alhagi maurorum* active ingredients and UC-related targets; (**B**) PPI network of intersection target; (**C**) *Alhagi maurorum* interferes with the “component-target-disease” interaction network of UC; (**D**) GO functional enrichment analysis on the shared targets between *Alhagi maurorum* and UC; (**E**) KEGG pathway enrichment analysis of the shared targets.

A network pharmacology approach was used to analyze the intersection of targets between *Alhagi maurorum* and UC. The results obtained from the database revealed 86 target proteins for *Alhagi maurorum* and 9395 target proteins for UC. Using the online program Draw Venn Diagram (https://www.bioinformatics.com.cn/static/others/jvenn/example.html, accessed on 15 January 2024), a Venn diagram was generated, which revealed 71 shared targets between *Alhagi maurorum* and UC (Figure 1A). A protein–protein interaction (PPI) network analysis of drug–disease targets was established, and the key targets were identified using the STRING database (Figure 1B). Figure 1B consists of 69 nodes and 888 edges. Conducting a topological analysis on the network, the degree values were used to reflect the size and color of target points. This analysis visualized the protein interactions among the shared targets of *Alhagi maurorum* and UC, which included tumor necrosis factor (TNF), interleukin-6 (IL-6), interleukin-1B (IL-1B), and other inflammation-related cytokines (Figure 1B,C). GO enrichment analysis revealed that the intersecting targets were involved in processes such as transmembrane tyrosine kinase receptor protein activity, the inflammatory response, and protein tyrosine kinase activity (Figure 1D). KEGG enrichment analysis revealed novel signaling pathways, including neuroactive ligand–receptor interactions, the calcium signaling pathway, serotonergic synapses, the cAMP signaling pathway, the cocaine addiction pathway, the CGMP-PKG signaling pathway, the alcoholism pathway regulating adipocyte lipolysis, GnRH secretion, folic acid resistance, and tyrosine metabolism (Figure 1E). *Alhagi maurorum* may alleviate inflammation in the body by inhibiting TNF cytokine protein expression, thus exerting a therapeutic effect on UC. The results indicate that *Alhagi maurorum* intervenes in UC by regulating TNF and NF-κB signaling pathways to inhibit inflammation and apoptosis, thereby exerting therapeutic effects.

### 2.1. Composition Analysis of Alhagi maurorum

**Figure 2:**
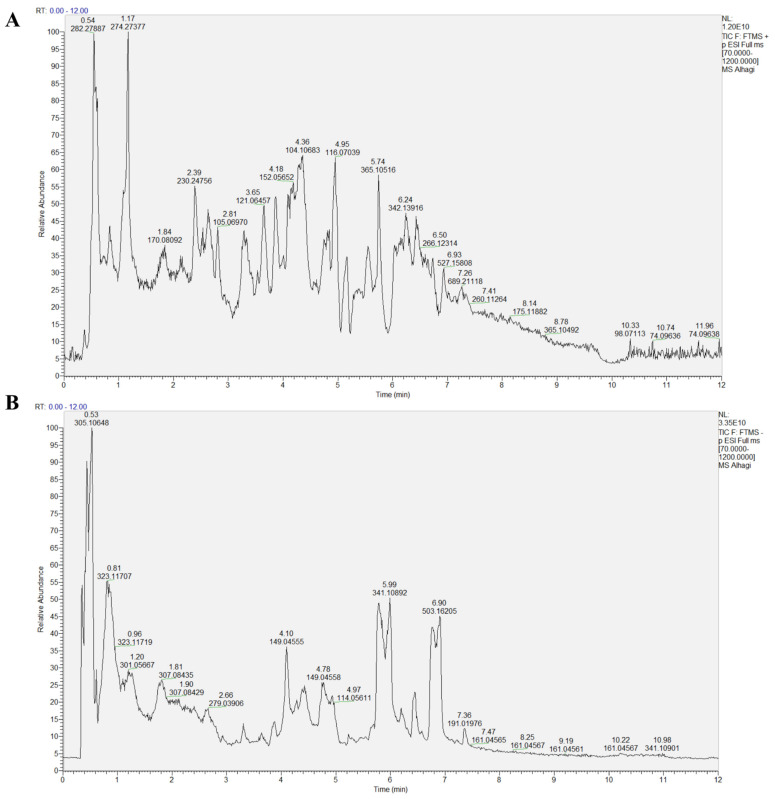
Chromatogram of the positive and negative ion modes of the *Alhagi maurorum* extract. (**A**) The base peak chromatograms of *Alhagi maurorum* in positive ion mode; (**B**) The base peak chromatograms of *Alhagi maurorum* in negative ion mode. Note: the horizontal axis in the figure represents the retention time of each chromatographic peak, while the vertical axis represents the intensity value of the peaks.

An LC-MS/MS (liquid chromatography tandem mass spectrometry) analysis was used for the identification of components in samples obtained from Alhagi maurorum water extraction. After the obtained mass spectrometry data were converted into the appropriate format, peak alignment, retention time correction, and peak area extraction were performed using the XCMD v1.12.0 program in MSDIAL software (http://prime.psc.riken.jp/compms/msdial/main.htm, accessed on 15 January 2024). Metabolite structure identification was conducted through accurate mass matching (<25 ppm) and secondary spectrum matching by searching public databases such as HMDB and MassBank. Figure 2 shows the total ion chromatograms of the samples in both positive and negative modes. The saccharides, carbohydrates and their derivatives, as identified by the experiment, were alpha-piperidinobutiophenone, beta-d-glucose, gamma-aminobutyric acid, and caryophyllene oxide: (4ar,5s)-9,9a-dihydroxy-3,4a,5-trimethyl-5,6,7,8,8a,9-hexahydro-4 h-benzo [1] benzofuran-2-one; 1,2-disinapoyl diglucoside; 1,2-disinapoyl diglucoside; and 1,2,3-benzenetriol (Figure 2 and Supplementary File S1 (Table S1)).

### 2.2. Differential Protein Expression between the MSC and Con Groups

**Figure 3:**
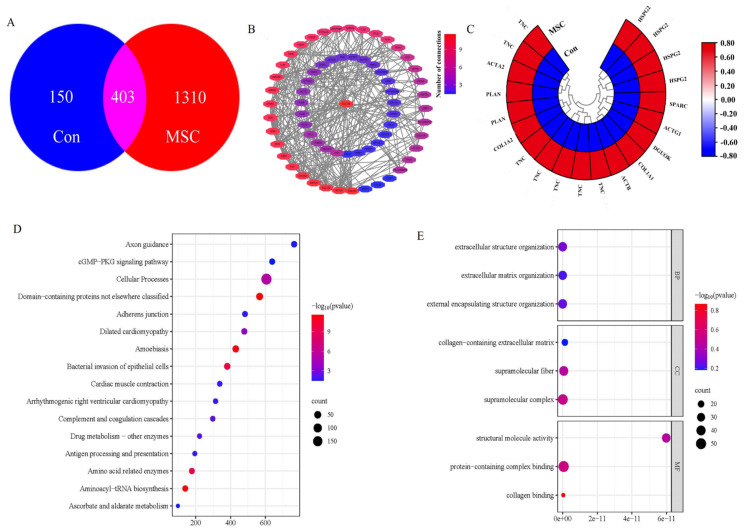
Differential protein expression between the MSC and control groups. (**A**) Venn diagram indicating the number of proteins in Con and MSC groups; (**B**) Protein–protein interaction (PPI) network of 66 most highly expressed proteins in MSC group (Supplementary File S2 (Figure S1)); (**C**) Heat map. The top 20 differentially expressed proteins in MSC group were selected; (**D**) KEGG functional annotation analysis; (**E**). GO functional annotation analysis.

To investigate the potential active components in MSC extracts, we analyzed the protein expression differences between the MSC and the control group through proteomic sequencing. The Venn diagram analysis revealed that there were 403 shared genes between the two groups, which may play similar biological roles or participate in the same biological processes. Notably, the number of unique proteins in the MSC group was 1310, indicating a richer diversity of protein expression in the MSC group. The MSC extract comprised a class of unique small-molecule proteins synthesized and secreted by stem cells. These proteins play a pivotal role in promoting the functional recovery of damaged tissues and cells (Figure 3A). To gain a deeper understanding of the interaction relationships among these proteins, we further constructed a protein–protein interaction (PPI) network with the top 66 proteins expressed highly in MSC group using Cytoscape v.3.6.1 software. As Figure 3B shows, COL1A1, COL3A1, ACTG1, GAPDH, COL1A2, and COL5A2 had a relatively high connection degree with other proteins. These proteins are capable of interacting with various growth factors, chemokines, enzymes, and other proteins. Interestingly, among these proteins, we found that some members of the collagen family were included, especially COL1A1, which was located at the core of the interaction network diagram. This indicated that collagen members may be important factors during the process of repairing tissue damage with stem cells (Figure 3B). To further compare the expression differences of these key proteins, we selected the top 20 proteins with the highest expression levels in the MSC group and conducted a heatmap analysis. Notably, we observed a significantly upregulated expression trend of key proteins, such as TNC, ACTA2, PLNA, COL1A2, and ACTB in the MSC group compared to the control group, indicating their potential crucial roles in the functions or characteristics of the MSC extract. These findings provide new insights and directions for further understanding the functions and regulatory mechanisms of the MSC extract, and their potential applications in the treatment of ulcerative colitis (Figure 3C). After a comprehensive analysis of the proteomic data, we identified significantly upregulated proteins in the MSC group involved in cellular processes and biological functions through KEGG analysis. They are mainly associated with core cellular processes, including cell proliferation, differentiation, migration, and signal transduction (Figure 3D). Additionally, through the application of the Gene Ontology (GO) approach, we observed an upregulated expression proteins in the MSCs groups closely related to protein-containing complex binding, supramolecular complexes, and supramolecular fiber signaling pathways (Figure 3E). The activation of these pathways might facilitate the MSC extract to better perform their specific biological functions, particularly in tissue repair and regeneration. These findings provide valuable insights for further exploring the roles of MSCs in the treatment of ulcerative colitis.

### 2.3. Alhagi maurorum and MSCs Ameliorate DSS-Induced Acute Colitis

**Figure 4:**
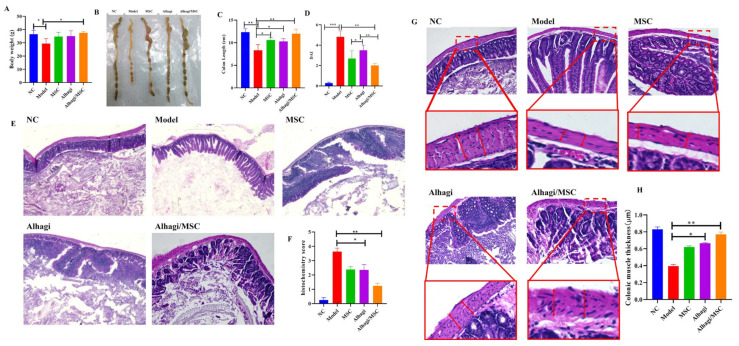
Effect of Alhagi/MSC extract on colonic histopathological morphology in mice with colitis. (**A**) Changes in body weight; (**B**) representative picture of colitis; (**C**) length of the colon; (**D**) DAI score of mice; (**E**) histopathological changes in colon tissues analyzed by hematoxylin and eosin (HE) staining (Scale bar = 200 μm); (**F**) histopathological observation and histological score of the mouse colon; (**G**,**H**) histopathological changes in colon muscle thickness (Scale bar = 200 μm); * *p* < 0.05; ** *p* < 0.01; *** *p* < 0.001.

To investigate the therapeutic effects of *Alhagi mauroru* and MSC extract on ulcerative colitis, BALB/c mice were intragastrically administered with *Alhagi mauroru* and MSC extract. As shown in Figure 4, during the experiment, the characteristics of the DSS-induced ulcerative colitis model mice included sustained weight loss and diarrhea. During the experiment, the weights of the mice were recorded, and changes in the weights of the mice were observed, as shown in Figure 4A. However, the administration of *Alhagi maurorum* and stem cell extracts significantly abrogated the decrease in body weight (*p* < 0.05). The colon length of the DSS group was 8.3 cm, which was significantly shorter than that of the normal group (*p* < 0.01), indicating that DSS had a damaging effect on the intestinal tract of the mice. The colon length of the mice in the MSC + model group was 10.3 cm. The colon length of the mice in the Alhagi extract + model group was 10.6 cm, while that of the mice in the + model group was 12 cm (Figure 4B,C). These results indicated that DSS had a damaging effect on the intestinal tract of mice, while the Alhagi/MSC extracts had a protective effect, indicating that the Alhagi/MSC extracts could improve ulcerative colitis in mice.

A statistical analysis was performed on the DAI scores of each group of mice, as shown in Figure 4D. The DAI score for the control group was 0.27 ± 0.43, while that for the DSS model group was 4.8 ± 0.96, indicating a significant difference between the two groups (*p* < 0.01). After intervention with Alhagi and MSCs, there was a significant decrease in DAI scores compared to the model group (*p* < 0.05), suggesting that Alhagi and MSCs effectively improved disease severity in mice. The DAI score for the Alhagi group was 3.4 ± 0.69, while that for the Alhagi/MSC group was 2.01 ± 1.37. Thus, it can be concluded that Alhagi/MSC can effectively alleviate symptoms such as weight loss, loose stools and bloody stools during colitis progression and has an anti-inflammatory effect on intestinal inflammation.

The histopathological score, which considers the degree of loss of structural integrity, the presence of crypts, crypt destruction, and mucosal surface changes, is a method used to estimate the degree of inflammation in the colon. Histological examination of the colon tissue of healthy mice revealed that the epithelial cells and crypts were structurally intact, the glands were neatly arranged, and there was no inflammatory cell infiltration. During ulcerative colitis in mice, neutrophils are recruited to the site of inflammation and can be found in the intestinal crypt and at the bottom of the ulcer, where they can form crypt abscesses. As shown in Figure 4E,F, colonic mucosal damage was significantly more common in UC model mice than in healthy control mice according to the typical colonic histology. DSS promotes extensive and severe ulceration of the epithelium. The colon of model mice with colitis exhibited structural disorders, irregular morphology, rupture of cryptic glands, severe damage to the surface epithelium, thickening of the submucosa (Figure 4E), mucosal ulceration, submucosal oedema, and intense infiltration of inflammatory cells. Mice given Alhagi and MSC extracts had a relatively intact surface epithelium, and Alhagi/MSC treatment reduced symptoms and relieved colon damage and inflammatory infiltration in mice with DSS-induced ulcerative colitis. Histological analysis revealed that the inner layer of the colon was structurally normal, as shown in Figure 4G; when observed under a 20-fold magnification, the model group exhibited a significant thinning of the colonic muscle layer compared to the NC group. The MSC and Alhagi groups showed an increase in muscle layer thickness after individual interventions, but their effectiveness in restoring muscle layer thickness was still inferior to that of the combined intervention with Alhagi/MSC (Figure 4G,H, *p* < 0.05). Many goblet cell crypts were preserved, and the inner layer was histologically similar to that of the colon in the healthy groups, indicating that the epithelium was repaired and that inflammatory response was reduced. The results of colonic histopathology in mice showed that the DSS-induced changes in colonic morphology were attenuated and improved by the combination of Alhagi and MSC extraction (Figure 4G,H). An analysis of colonic tissue morphology in mice revealed significant mucosal damage in ulcerative colitis compared to the representative colon histology of the model and healthy control groups. DSS administration promoted extensive and severe epithelial ulcers, resulting in severe disruption of thecolonic structure. However, treatment with Alhagi/MSC extract alleviated goblet cell necrosis, with only minimal inflammatory cell infiltration observed.

### 2.4. Analysis of Gut Microbiota Diversity

**Figure 5:**
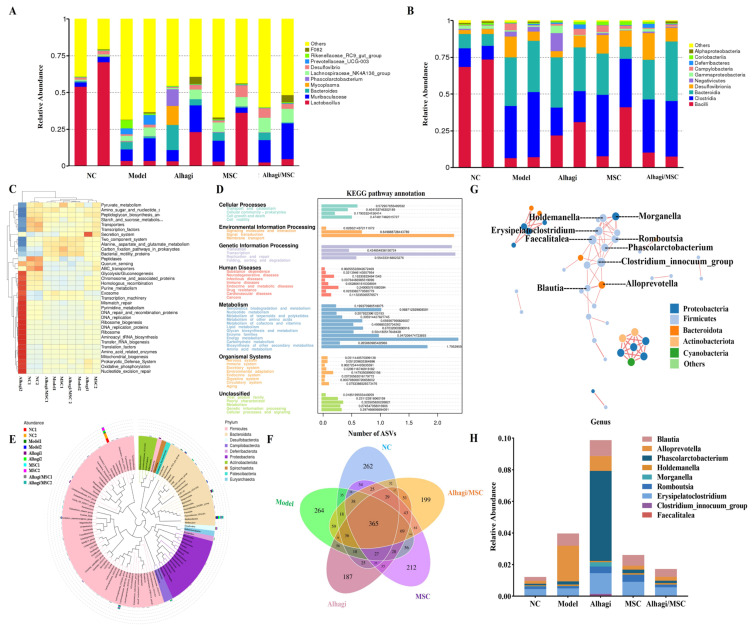
Gut microbiota analysis upon Alhagi/MSC treatment in colitis model mice. (**A**) Community column diagram at the genus level; (**B**) community column diagram at the order level; (**C**) clustering map of species abundance; (**D**) KEGG analysis; (**E**) phylogenetic tree of representative species sequences in genus level (Supplementary File S2 (Figure S2)); (**F**) Venn diagram indicating ASVs number in five groups; (**G**) the dynamic network diagram. It shows the relationship between the interactions of different bacterial genera. The different color represents the different phylum. Each circle represents a genus of bacteria, and the lines between the circles represent their interactions; (**H**) The bar graph presents the abundance of these bacterial genera in each group. According to the dynamic network diagram, the top 9 bacterial genera with the strongest interaction were selected.

To determine whether Alhagi and MSC extract modulate the gut microbiota during UC treatment, we performed a 16S rRNA sequencing analysis on stool samples from the mice. The histogram of the gut microbiota structure displays the microbial species and their relative abundance at the phylum level. Compared to the DSS-treated group, supplementation with Alhagi/MSC upregulated the abundance of the Lachnospiraceae_NK4A136_group and downregulated the abundance of Muribaculaceae and Prevotellaceae UCG-003 (Figure 5A). Figure 5B shows the relative abundance of gut microbiota at the genus level in the different treatment groups. The relative abundances of Campylobacteria, Clostridia, Desulfovibrionia, Lachnospiraceae_NK4A136_group, and Negativicutes significantly changed in the colonic contents of DSS-induced colitis mice; however, their abundances were significantly reduced after the oral administration of prickly sugar and stem cell extract. Additionally, supplementation with prickly Alhagi and MSC group significantly increased the relative abundances of Bacteroidia, I Alphaproteobacteria Romboutsia, Lactobacillus, Allobaculum, norank_f_norank_o_Clostridia_UCG-014, and Odoribacter (Figure 5B). Clustering heatmap analysis of flora species abundance among five groups is shown in Figure 5C. We further utilized PICRUSt to infer the composition of microbial functional genes in the sequenced samples based on the species composition obtained from 16S sequencing, predicted the expression levels of relevant pathways, and analyzed inter-group functional differences. The significant differences in abundance between two groups at Level_3 of KEGG pathways is represented using error bar plots, as shown in Figure 5D. These include transport and catabolism, cellular community-prokaryotes, cell growth and death, and cell motility. As shown in Figure 5E, the phylogenetic tree visually demonstrates the significant changes in the associated microbial communities of Firmicutes, Bacteroidetes, Desulfurobacteria, and Campylobacter in each group. Firmicutes and Bacteroidetes were the dominant microbial groups in most groups, but their relative abundance varied among groups. To further delve into the community analysis, we plotted a Venn diagram. Through a detailed comparison, we discovered that there are 365 Amplicon Sequence Variants (ASVs) shared among the control group, model group, Alhagi group, MSC group, and Alhagi/MSC group (Figure 5F). Among these shared ASVs, there may exist a core microbiota representative of a specific environment or condition, playing a crucial role in maintaining the homeostasis of the gut microbiota. To gain a clearer understanding of the associations among microbial genera across different groups, we selected the top 40 microbial genera for the calculation of Spearman correlation coefficients. Based on these calculations, we identified connections with correlation coefficients greater than 0.6 or less than −0.6 and significant (*p*-value < 0.05) as effective connections, which were then used to construct a dynamic network diagram. We further narrowed down our focus to the nine genera with the highest number of effective connections, which were, respectively, Faecalitalea, Clostridium_innocuum, Erysipelatoclostridium, Romboutsia, Morganella, Holdemanella, Phascolarctobacterium, Alloprevotella, and Blautia, identifying them as our predicted target genera. We hypothesize that extracts from Alhagi and MSC may alleviate US by modulating these microbial genera (Figure 5G). To validate this hypothesis, we further examined the abundance of these predicted target genera in the control group, model group, Alhagi group, MSC group, and Alhagi/MSC group. Our results showed that the relative abundances of Clostridium_innocuum, Holdemanella, Phascolarctobacterium, Alloprevotella, and Blautia increased in the model group compared to the control group, while the abundances of Romboutsia and Morganella decreased. Notably, the Alhagi group, MSC group, and Alhagi/MSC group reversed these changes to varying degrees. Therefore, we believe that these microbial genera are key players in the treatment of UC with Alhagi and MSC extracts (Figure 5H).

### 2.5. Effects of MSCs and Alhagi maurorum Extracts on Colonic RNA Levels in Mice

**Figure 6:**
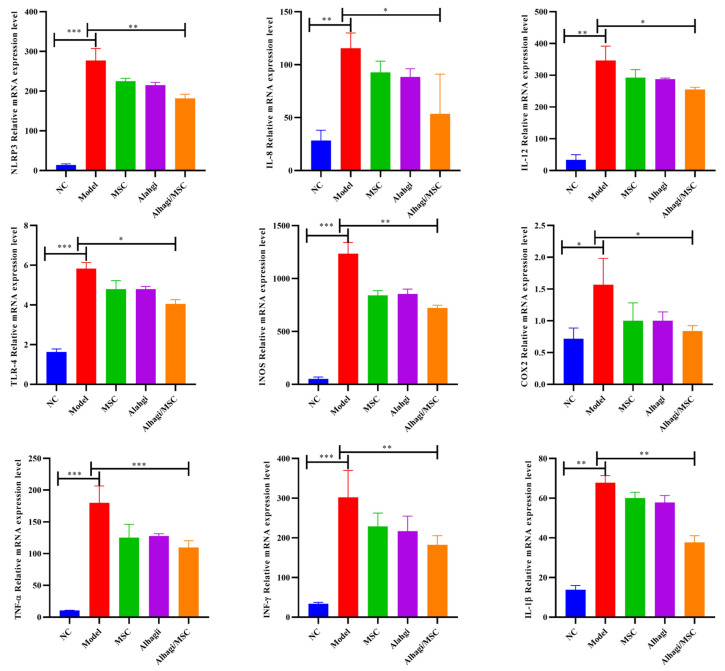
Effect of Alhagi/MSC on the RNA levels of NLRP3 inflammasome signaling pathway members in the colons of mice. * *p* < 0.05; ** *p* < 0.01; *** *p* < 0.001.

As the severity of ulcerative colitis is positively correlated with the levels of certain inflammatory mediators and the production of proinflammatory cytokines in the inflamed colon, we mainly focused on the abnormal expression of TNF-α, INF-γ, IL-1β, IL-8, IL-12, NOS, and TLR-4, which are well-known inflammatory markers that play important roles in the pathogenesis of ulcerative colitis. The production of proinflammatory mediators plays a key role in DDS-induced ulcerative colitis. An RT–qPCR analysis was performed to investigate the effects of MSCs and Alhagi extract on the gene expression of inflammation-related genes (Figure 6).

Type I interferons, including INF-γ and INF-α, have been used for several years to inhibit NLRP3 and other inflammatory bodies in various autoimmune and autoinflammatory diseases, and INF-γ was significantly upregulated in ulcerative colitis (*p* < 0.001) to activate the NLRP3 inflammasome, which promotes inflammation. The expression of inflammatory factors was significantly upregulated in the colon tissues of DSS-induced ulcerative colitis model mice (*p* < 0.05). In contrast, MSCs and Alhagi extracts (Figure 6; *p* < 0.001) significantly inhibited the expression of cytokines and chemokines in colon tissue and the transcription of a series of inflammatory signaling molecules. Additionally, the effects of MSC and Alhagi extracts on ulcerative colitis were somewhat protective.

**Figure 7:**
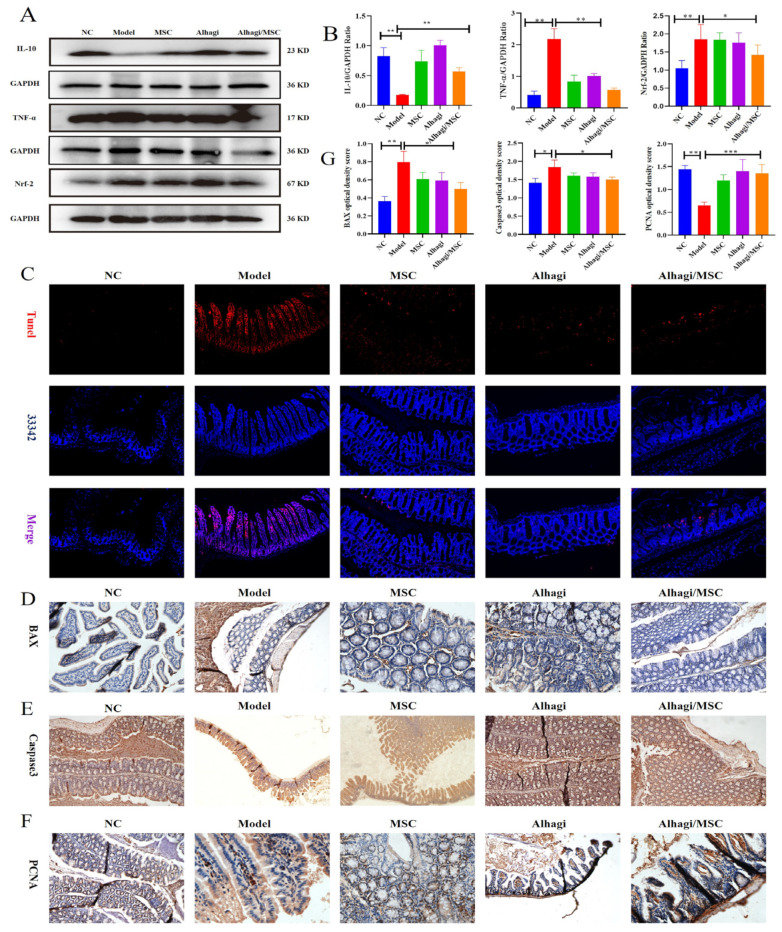
The effect of Alhagi/MSC extracts on apoptosis- and inflammation-related molecules in the colon tissue of colitis model mice. (**A**,**B**) Expression levels of the IL-10, TNF-α, and Nrf-2 in the colon tissues of colitis model mice; (**C**) TUNEL staining (Scale bar = 100 μm); (**D**–**F**) immunocytochemical analysis of BAX, Caspase3, and PCNA; (**G**) quantitative analysis of immunohistochemical staining of BAX, Caspase3, and PCNA in mice intestinal tissue (Scale bar = 200 μm). * *p* < 0.05; ** *p* < 0.01; *** *p* < 0.001.

To investigate whether stem cell/Alhagi extracts alleviates colitis by regulating the NLRP3 signaling pathway, Western blot and immunohistochemical analysis of NLRP3 pathway-associated protein in colon tissue were performed. In the present study, DSS-treated mice exhibited significantly increased TNF-α, IL-10, Nrf-2 (Figure 7A,B; *p* < 0.05), and BAX protein levels compared with the NC group (Figure 7D,G), suggesting that the NLRP3 inflammasome was activated and pyroptosis occurred in the colon tissue. On the contrary, Alhagi/MSC significantly downregulated the expression of apoptosis and inflammasome-related proteins, such as NLRP3, BAX, and Caspase3 levels, and weakened the TUNEL positive signal in DSS-induced mouse colitis models (Figure 6 and Figure 7C,G), while it increased the PCNA expression levels (Figure 7F,G). This meant that Alhagi/MSC reduced the apoptosis and inflammation-related molecular expression in colon tissues, while promoting proliferation-related molecular expression. These results indicated that the Alhagi/MSC effectively improved DSS-induced UC by inhibiting the activation of the NLRP3 inflammasome to prevent the occurrence of pyroptosis, and decreased apoptosis in the colon tissue. Alhagi/MSC may protect the colonic barrier in mice by preserving tight-junction proteins, and may alleviate dextran sulfate sodium (DSS)-induced ulcerative colitis in mice by inhibiting the abnormal activation of the NLRP3 inflammasome pathway and TNF signaling pathway, thereby reducing colon damage.

## 3. Discussion

Due to their ability to regenerate, differentiate, and modulate the immune system, stem cells have potential in the treatment of numerous diseases. However, the limited migration of transplanted MSCs to specific tissues poses a significant challenge that must be addressed before MSCs can be effectively utilized as a therapeutic approach for diverse medical conditions [16]. The research has indicated that the paracrine mechanism of MSCs may be the primary factor contributing to their promising therapeutic effects [16,17]. In comparison to directly transplanting MSCs, it has been demonstrated that exosomes derived from MSCs exhibit superior therapeutic effects in various diseases, including renal fibrosis and chronic cutaneous wounds [18,19]. The oxygen concentration plays a crucial role in ensuring the survival of MSCs. Adipose-derived mesenchymal stem cells (AD-MSCs) are derived from the bone marrow cavity, where the oxygen concentration typically ranges from 1% to 7%. Therefore, when cultured in low oxygen conditions, ADMSCs are able to better adapt to the hypoxic tissue environment that occurs during inflammation [16,17,20]. A study demonstrated that hypoxia can enhance the functionality and movement of ADMSCs while preventing their programmed cell death. Hence, we aimed to investigate whether ADMSC-Exos exhibit enhanced therapeutic effects compared to those under normal oxygen conditions. To address this query, a series of in vivo experiments were conducted. The results revealed that treatment with ADMSCs facilitated the healing process of intestinal mucosal injury in patients with UC and further showed that combining MSC/Alhagi treatment yielded superior outcomes compared to the other available treatments.

During the development of UC, a series of colonic pathological changes related to inflammation will occur, manifested as weight loss, rectal bleeding, edema, ulcers, shortened colon length, and increased DAI scores and histological scores [21,22]. However, supplementation with Alhagi and MSC can significantly improve the aforementioned symptoms of DSS-induced colitis. An impaired intestinal mucosal barrier function is closely associated with the occurrence of UC, characterized by sparse and shortened intestinal villi, widened intercellular spaces, severe mucosal damage, loss of goblet cells, decreased mucus production, crypt distortion, and excessive neutrophil infiltration. Nevertheless, supplementation with Alhagi and MSC can effectively prevent intestinal mucosal damage, recover mucosal ultrastructure, promote mucus secretion, and increase goblet cell numbers. It has been reported that the reduced expression of tight-junction proteins leads to increased intestinal permeability, bacterial translocation, and a further exacerbation of inflammatory response. In this study, supplementation with Alhagi and MSC can effectively enhance the expression of PCNA, restore BAX and Tunel in colon tissue (*p* < 0.05), and thus achieve improvements in UC.

The development of UC is usually accompanied by a decrease in gut microbiota diversity, characterized by the loss of beneficial bacteria and an increase in harmful bacteria [23,24,25,26]. However, the oral administration of Alhagi and MSC not only increases the diversity of intestinal microbiota in UC mice but also alters the structure of the gut microbiota. Furthermore, it has been shown that Turicibacter, Enterococcus, Parabacteroides, Parasutterella, Erysipelatoclostridium, and Acinetobacter are mainly enriched in the DSS group. These bacteria exhibit higher relative levels in UC and are closely associated with the progression of colitis. It has been reported that Crohn’s disease also shows a higher infection rate for Helicobacteraceae. However, the oral administration of Alhagi and MSC can not only reduce the abundance of Helicobacteraceae in intestinal microbiota but can also increase the relative abundance of Romboutsia, Lactobacillus, and Odoribacter. These are considered beneficial for the production of short-chain fatty acids via the gut microbiota metabolism and have preventive and inhibitory effects on inflammatory reactions, thus playing an important role in maintaining intestinal immune homeostasis. In addition to this effect, the oral administration of Alhagi and MSC increases SCFA levels in feces from UC mice, which plays a certain role in maintaining the intestinal microenvironment stability. Therefore, Alhagi and MSC may exert their anti-UC effects through regulating specific gut microbial communities and their metabolites.

The DSS-induced ulcerative colitis model is widely adopted due to its simplicity, low cost, high success rate, and similarity to the clinical symptoms, lesion sites, histopathological manifestations, and treatment response of human UC [27,28,29]. The mechanism by which DSS induces colitis is not fully understood at present; however, studies have found that mice drinking DSS can exert a toxic effect on the colonic epithelial barrier, disrupting the intestinal barrier function and increasing permeability. As a result, macromolecules can penetrate the mucosal tissue and trigger abnormal immune cell reactions, leading to inflammation. Additionally, the anticoagulant properties of DSS itself may contribute to colon bleeding in mice. Under normal circumstances, following experimental protocols, mice freely consume 2–5% DSS dissolved in water and develop acute or chronic intestinal inflammation. After successful induction using the DSS modeling approach in mice, weight loss, diarrhea, mucus, and bloody stools are observed, along with colonic mucosal damage such as swelling or atrophy of crypt structures and increased levels of inflammatory factors (*p* < 0.05). In DSS-induced colitis, specifically, immunocyte dysfunction leads to abnormal secretion levels of pro-inflammatory cytokines such as tumor necrosis factor-alpha, interleukin-1β, and IL-6, while anti-inflammatory cytokines like IL-10 secretion decrease (*p* < 0.01). C57BL/6 adult mice treated with 3% DSS solution were observed for symptoms on the 3rd, 5th, 7th, and 10th days, revealing the onset of colitis symptoms from day 3 onwards, worsening after 5 days, and reaching peak pathological changes by day 7, characterized by decreased body weight, bloody stools, damage to crypt structures in the colon, and the infiltration of large numbers of mainly neutrophil-dominated inflammatory cells.

According to this, the present study established a colitis model in mice by allowing them to freely drink a 3% DSS solution for 7 days. The above study found consistent results with our experimental findings. During the modeling period, except for the normal group of mice, DSS-induced mice showed a decrease in body weight from day 3 until the end of the experiment, and their DAI continuously increased until day 9, indicating that the mice exhibited symptoms of colitis during modeling. After the experiment ended, a pathological evaluation of colon tissues revealed colon atrophy in colitic mice. HE histopathological sections showed the severe destruction of crypt structures and infiltration of inflammatory cells. Additionally, the spleen index significantly increased while the thymus index significantly decreased in colitic mice (*p* < 0.05). The spleen and thymus are closely related to immune cell development, differentiation, maturation, and immune response regulation, thus suggesting that DSS induction caused immune abnormalities in mice. Furthermore, it was found that pro-inflammatory cytokines IL-6 and TNF-α secretion significantly increased while anti-inflammatory cytokine IL-10 levels decreased in colitic mice’s serum. This confirms that DSS induction leads to immune abnormalities and inflammation reactions in mice. The findings also indicate that DSS induces the disruption of normal structure in colon tissues, as well as an increase in pathological scores, along with a significant elevation of pro-inflammatory cytokine content relative mRNA expression, and protein levels.

However, compared with DSS-induced colitis mice, the oral supplementation of MSC or Alhagi reduced intestinal inflammatory responses, including weight gain, decreased DAI scores, alleviated colon tissue damage, and improved immune organ indices regulating inflammatory cytokines. Furthermore, the effect of Alhagi/MSC on colitis was found to be dose-dependent. Similar to our findings, some plant-derived oligosaccharides or milk-derived oligosaccharides also demonstrated anti-inflammatory and immunomodulatory functions in gastrointestinal diseases. It was discovered that α-lactose could exhibit protective properties against colitis in mice by reducing the colon shortening degree, and mitigating colon tissue damage and inflammatory cell infiltration, as well as decreasing the expression of inflammatory factors such as NLRP3, IL-6, COX-2, IL-1β, and TNF-α in colon tissues. The study found that Alhagi/MSC significantly reduced DSS-induced mice’s DAI scores, and the histological damage scores and expression levels of IL-6 and TNF-α in colon tissues, while improving intestinal inflammatory response. Additionally, adding an appropriate proportion of MSC to the diet increased body weight, induced by DSS and lowered pathological scores in colon tissues for rats. From the above analysis, we can conclude that Alhagi or MSC can reduce DSS-induced intestinal inflammation response and protect host health. These results suggested that the ameliorative effect of MSC/Alhagi on DSS-induced colitis may be closely related to the inhibition of proinflammatory cytokine release involving NLRP3 signaling pathways.

## 4. Materials and Methods

### 4.1. Experimental Ulcerative Colitis and Treatment

Six-week-old male BALB/c mice were obtained from Xi’an Hangsi Biological Technology Co., Ltd. (Xi’an, China). The mice were housed in individually ventilated caging systems under a 12 h light/dark cycle at a temperature range of approximately 21–25 °C and humidity levels of around 50–60%. They were provided with unrestricted access to sterilized standard rodent chow and water. All animal experiments that were conducted adhered to the regulations set by Chinese legislation on laboratory animal use and care and received approval from the ethics committee of Northwest A & F University (Approval No. NWAFU202207010).

After a period of adaptive feeding, lasting 7 days, the mice were randomly divided into five groups (n = 12 for each group): the control group with normal conditions (referred to as the NC group), a group induced with UC using DSS (referred to as the DSS group), a UC treatment group receiving water extract of *Alhagi maurorum* (referred to as Alhagi group), a UC treatment group receiving mesenchymal stem cells (referred to as the MSC group), and a combined treatment group receiving both mesenchymal stem cells and *Alhagi maurorum* extract for UC (referred to as the Alhagi/MSC group). Except for the NC group, all other groups were given drinking water supplemented with 3% DSS in distilled water for 7 days to induce colitis. Subsequently, they were provided with regular drinking water for a recovery period of 6 days. The mice in the Alhagi/MSC groups received daily oral administration of a saline solution containing dissolved extracts of *Alhagi maurorum* and mesenchymal stem cells at a dosage of 300 mg/kg BW/day, over a span of 12 days. In addition, the control group and DSS group mice were orally administered with an equal volume of saline throughout the entire duration of the experiment, while being fed a standard diet. Daily observations were made of body weight, stool consistency, the overall condition of the mice, and any presence of visible blood in both the feces and around the anus. These observations were used to calculate the disease activity index. Following the collection of feces and blood samples, euthanasia was performed via cervical dislocation, and colons were immediately removed for the measurement of colon length. The colons were then preserved for histological examination and RNA extraction purposes.

### 4.2. Histopathological and Immunohistochemical Analyses

The colon tissue was fixed in a 4% solution of paraformaldehyde for a duration of 48 h. Following fixation, the tissue was embedded in paraffin and subsequently sectioned. To enable microscopic observation, the sections were stained using hematoxylin and eosin (H&E) solution. The histological score was determined utilizing established methods, with injury severity assessed based on the extent of inflammatory cell infiltration, mucosal injury, and crypt injury.

The BAX/PCNA/Cas3 protein expression in colon tissue was assessed through immunohistochemical staining, following established protocols. In brief, the colon sections were subjected to 3% hydrogen peroxide treatment for 10 min and then incubated with a primary antibody (1:300) overnight at 4 °C. This was followed by incubation with a secondary antibody at room temperature for 2 h. The resulting images were observed under an Olympus microscope, where positive staining was shown using a brown color while counterstained nuclei were blue. ImageJ v.1.46r software was used to quantify the average levels of BAX/PCNA/Cas3 in colon tissues as the percentage of positive areas in each image.

### 4.3. Measurement of Inflammatory Cytokines

The serum samples were analyzed for TNF-α, IL-1β, and IL-6 levels using ELISA kits (manufactured in Nanjing, China). The experimental protocols followed the instructions provided by the manufacturer.

### 4.4. Analysis of the Gut Microbiota

Fecal samples were processed using the TIANamp Stool DNA Kitto (CAS No. 4992199, TIANGEN, Beijing, China) to extract genomic DNA. The purity of the DNA was assessed through 1% agarose gel electrophoresis. Operational taxonomic units (OTUs), based on a 97% sequence similarity threshold, were determined using QIIME v.2023.12 software. Taxonomic analysis was performed by importing these OTUs into RDP algorithm v.715a436f software with a confidence threshold set at 0.7. Estimation of α diversity was carried out utilizing Mothur software version 1.30.2, while Principal Coordinate Analysis (PCoA) and hierarchical clustering analysis employed representative sequences of OTUs based on Bray–Curtis distance. A Venn diagram was created, and R v.4.2.3. software was utilized to generate a hierarchical clustering heatmap. Moreover, the linear discriminant analysis (LDA) effect size algorithm was employed to differentiate the key OTUs representing differences among the experimental groups of mice.

### 4.5. Screening of Active Ingredients and Target Genes of Alhagi maurorum

Using “*Alhagi maurorum*” as a keyword, we searched the Traditional Chinese Medicine Systems Pharmacology Database and BATMAN TCM Analysis Platform database to identify the bioactive components and target proteins of this herb. We filtered the results based on the oral bioavailability (≥30%) and drug likeness (≥0.18) criteria to obtain the main active ingredients and their corresponding targets. Perl scripts were used to obtain the gene names. Using ‘UC’ as a keyword, we searched the Online Mendelian Inheritance in Man and GeneCards databases to determine the target genes associated with these conditions. R language was used to create a script that compared the disease-related targets with those of *Alhagi maurorum*, generating a Venn diagram to obtain their common targets. The obtained key/target proteins were input into the DAVID database to retrieve relevant data on biological processes (BP), cellular components (CC), and molecular functions (MF), as well as related pathways from the KEGG database ranked by *p* values.

### 4.6. Western Blot Analysis

Protein was extracted from colon tissue using a radioimmunoprecipitation assay (RIPA) lysis buffer. The primary antibodies were Interleukin-10 (IL-10), tumor necrosis factor-α (TNF-α), NF-E2-related factor 2 (Nrf-2, 1:1200, Immunoway Biotechnology, Plano, TX, USA), glyceraldehyde phosphate dehydrogenase (GAPDH, 1:3000), and the secondary antibodies conjugated to horseradish peroxidase (1:3000, Immunoway Biotechnology). The other proteins were normalized using GAPDH.

### 4.7. Statistical Analysis

The data are expressed as the mean ± SEM. All the graphing and statistical analyses were performed using Prism GraphPad v.8.0 software. Multiple groups were compared using one-way ANOVA, followed by the least significant difference post hoc test, and *p* < 0.05 was considered to indicate statistical significance.

## 5. Conclusions

In conclusion, mesenchymal stem cells and *Alhagi maurorum* extract may protect the colon barrier by protecting colonic mucosa tight-junction proteins, and may alleviate DSS-induced colitis in mice by inhibiting the abnormal activation of the NLRP3 inflammasome signaling pathway and downregulating apoptosis pathway-related proteins.

## Data Availability

All data generated and analyzed during this study are included in this published article.

## Supplementary Materials

The following supporting information can be downloaded at: https://www.mdpi.com/article/10.3390/ijms25073653/s1.
