# Supplementary material for: Protective Effect of Mesenchymal Stem Cell Active Factor Combined with Alhagi maurorum Extract on Ulcerative Colitis and the Underlying Mechanism
Source: Int J Mol Sci. 2024 Mar 25;25(7):3653. doi: 10.3390/ijms25073653 (PMC11011388; doi:10.3390/ijms25073653)
Supplement: Supplementary file 1 [file ijms-25-03653-s001.zip › ijms-2850990-supplementary S1.pdf]

International Journal of Molecular Sciences

MDPI

Supplementary File S1

Composition analysis of Alhagi maurorum

Grind the prepared sample with liquid nitrogen, accurately weigh 100 mg of the ground sample and transfer it into a beaker containing 200 μl pre-cooled water and 800 μl pre-cooled methanol/acetonitrile (1:1) for ice bath sonication. Sonicate the mixture for 60 minutes, then remove the sample and incubate it at -20°C for 1 hour to precipitate proteins. After precipitation, centrifuge at 16,000 g for 20 minutes and transfer the supernatant. Dry the obtained solution using a high-speed vacuum concentrator and store it for future use. When ready for mass spectrometry analysis, retrieve the sample from storage and add an additional 100 ml acetonitrile-water solution (1:1) to resuspend it in the container. Centrifuge again at 16,000 g for 15 minutes before injecting the supernatant into analytical instruments, the determination of the composition of Alhagi maurorum. identification was conducted through accurate mass matching and secondary spectrum matching by searching public databases such as HMDB and MassBank. Figure 2 shows the total ion chromatograms of the samples in both positive and negative modes. The saccharides, carbohydrates and their derivatives, as identified by the experiment, were alpha-piperidinobutiophenone, beta-d-glucose, gamma-aminobutyric acid, and caryophyllene oxide: (4ar,5s)-9,9a-dihydroxy-3,4a,5-trimethyl-5,6,7,8,8a,9-hexahydro-4 h-benzo [1] benzofuran-2-one; 1,2-disinapoyl diglucoside; 1,2-disinapoyl diglucoside; and 1,2,3-benzenetriol (Table S1).

Table of chemical constituents of *Alhagi maurorum*

ID	m/z	rt(s)	PPM	Name	Precursor_Type	score	Class	SubClass	rtmax
**M441T61**	441.1227369	61.0639	0.728874784	.alpha.-apooxytetracycline	[M-H]-	0.883448085			61.5627
**M241T316**	241.0119923	316.4325	0.552617176	.alpha.-d-galactose 1-phosphate	[M-H-H2O]-	0.9538305267792 27	Organooxygen compounds	Carbohydrates and carbohydrate conjugates	318.593
**M795T237**	795.4541195	236.72	0.437868059	.alpha.-hederin	[M+HCO2]-	0.796891184794032	Prenol lipids	Triterpenoids	237.229
**M147T78**	147.0297585	77.9405	1.761572857	.alpha.-keto-.gamma.-(methylthio)butyric acid	[M-H]-	0.940244229	Fatty Acyls	Fatty acids and conjugates	78.9931
**M203T247_1**	203.0673661	247.443	0.26845661	.alpha.-L-Glu-Gly	[M-H]-	0.848433216783731	Carboxylic acids and derivatives	Amino acids, peptides, and analogues	248.081
**M194T27**	194.0822809	27.06985	0.604033885	.alpha.-methyl-l-tyrosine	[M-H]-	0.948542008087599			30.0613
**M421T51**	421.0812506	50.9833	0.928435946	.alpha.,.alpha.'-trehalose 6-phosphate	[M-H]-	0.723047997	Organooxygen compounds	Carbohydrates and carbohydrate conjugates	52.2925
**M359T153**	359.1350518	153.171	0.355970638	.beta.-d-allose	[2M-H]-	0.849712886	Organooxygen compounds	Carbohydrates and carbohydrate conjugates	154.993

**M502T281**	502.2334201	281.008	1.231609362	.beta.-d-glucopyranosiduronic acid, 5-[3-[(2,2,3,3-tetramethylcyclopropyl)carbonyl]-1h-indol-1-yl]pentyl	[M-H]-	0.968871820193026	Fatty Acyls	Fatty acyl glycosides	282.337
**M351T255**	351.1298806	254.672	0.366220747	.beta.-estradiol 3-sulfate	[M-H]-	0.759110575315624	Steroids and steroid derivatives	Sulfated steroids	254.877
**M527T306**	527.1808875	306.303	1.128040542	.beta.-estradiol-3-glucuronide-17-sulfate	[M-H]-	0.820877665	Steroids and steroid derivatives	Steroidal glycosides	307.275
**M89T200**	89.02455489	199.835	0.982026418	.beta.-hydroxypropionic acid	[M-H]-	0.998196435028262	Hydroxy acids and derivatives	Beta hydroxy acids and derivatives	200.83
**M249T245**	249.0616328	244.8425	1.097836093	.gamma.-glu-cys	[M-H]-	0.919689700839694	Carboxylic acids and derivatives	Amino acids, peptides, and analogues	256.175
**M168T182**	168.1030611	181.936	0.17400961	(-)-.alpha.-kainic acid	[M-H-CO2]-	0.897139424720396	Carboxylic acids and derivatives	Amino acids, peptides, and analogues	182.037
**M289T422**	289.0565428	421.5585	0.621819197	(-)-catechin	[M-H]-	0.946716858197139	Flavonoids	Flavans	429.59
**M289T89**	289.0719452	89.3151	0.325664193	(-)-epicatechin	[M-H]-	0.964875132469936	Flavonoids	Flavans	91.1058
**M305T227**	305.0701711	227.451	0.490061966	(-)-epigallocatechin	[M-H]-	0.900071119380277	Flavonoids	Flavans	227.764
**M171T121**	170.9967147	120.519	1.252765295	(-)-hydroxycitric acid lactone	[M-H-H2O]-	0.978205561	Carboxylic acids and derivatives	Tricarboxylic acids and derivatives	121.734
**M411T182**	411.1326618	181.992	1.541172252	(-)-riboflavin	[M+Cl]-	0.713641908562806	Pteridines and derivatives	Alloxazines and isoalloxazines	182.146
**M263T111**	263.1290411	111.11	0.333354345	(+)-abscisic acid	[M-H]-	0.986306107243907	Prenol lipids	Sesquiterpenoids	112.613
**M357T130**	357.134349	129.603	0.636017885	(+)-pinoresinol	[M-H]-	0.98678558357028			130.947
**M348T194**	348.1931225	194.193	1.042498077	(1-(5-fluoropentyl)-1h-indazole-3-carbonyl)-l-valine	[M-H]-	0.805152401330052	Carboxylic acids and derivatives	Amino acids, peptides, and analogues	194.803
**M495T250**	495.1908668	249.625	0.384397839	(1r,4s,7r,8s)-7-[(2e,4e)-hexa-2,4-dienoyl]-3-hydroxy-8-[(2s)-3-hydroxy-2,4-dimethyl-5-oxo-2,5-dihydrofuran-2-yl]-5-[(2e,4e)-1-	[M-H]-	0.891494973	Dihydrofurans	Furanones	250.035

				hydroxyhexa-2,4-dien-1-ylidene]-1,3-dimethylbicyclo[2.2.2]octane-2,6-dione					
**M425T25**	425.2580283	25.4587	0.101953658	(2e,6e,10e)-13-[(2r)-6-hydroxy-2,8-dimethyl-3,4-dihydrochromen-2-yl]-2,6,10-trimethyltrideca-2,6,10-trienoic acid	[M-H]-	0.967549249	Prenol lipids	Quinone and hydroquinone lipids	37.4765
**M521T187**	521.2031691	186.577	0.504917383	(2r,3r,4s,5s,6r)-2-[[7-hydroxy-1-(4-hydroxy-3-methoxyphenyl)-3-(hydroxymethyl)-6-methoxy-1,2,3,4-tetrahydronaphthalen-2-yl]methoxy]-6-(hydroxymethyl)oxane-3,4,5-triol	[M-H]-	0.823996953			186.906
**M577T473**	577.1991793	473.413	0.206973491	(3s,4s)-5-[(3s,4s)-4,10-dihydroxy-7,9-dimethoxy-3-methyl-3,4-dihydro-1h-benzo[g]isochromen-5-yl]-7,9-dimethoxy-3-methyl-3,4-dihydro-1h-benzo[g]isochromene-4,10-diol	[M-H]-	0.807136012			474.882
**M497T61**	497.2066543	60.67585	0.480714395	(5r)-5-[(1s,2s,3r,4r)-3-[(4e)-hex-4-enoyl]-6-[(2e,4e)-hexa-2,4-dienoyl]-5,7-dihydroxy-4,7-dimethyl-8-oxobicyclo[2.2.2]oct-5-en-2-yl]-4-hydroxy-3,5-dimethyl-2,5-dihydrofuran-2-one	[M-H]-	0.988985764659595	Organooxygen compounds	Carbonyl compounds	70.3225
**M525T199**	525.1651494	199.1825	1.615331807	(5z)-4-[2-[2-(3,4-dihydroxyphenyl)ethoxy]-2-oxoethyl]-5-ethylidene-6-[(2s,3r,4s,5s,6r)-3,4,5-trihydroxy-6-(hydroxymethyl)oxan-2-yl]oxy-4h-pyran-3-carboxylic acid	[M-H]-	0.888991243406312	Prenol lipids	Terpene glycosides	199.778

**M241 T43**	241.002463	42.9281	0.299434709	(e)-1-hydroxy-2-methylbut-2-enyl 4-diphosphate	[M-H-H2O]-	0.967519850684911			45.2018
**M735 T463**	735.2203955	463.351	1.069952869	(phenoxymethyl)penicilloic acid	[2M-H]-	0.923876122	Carboxylic acids and derivatives	Amino acids, peptides, and analogues	463.69
