# Supplementary material for: Protective Effect of Mesenchymal Stem Cell Active Factor Combined with Alhagi maurorum Extract on Ulcerative Colitis and the Underlying Mechanism
Source: Int J Mol Sci. 2024 Mar 25;25(7):3653. doi: 10.3390/ijms25073653 (PMC11011388; doi:10.3390/ijms25073653)
Supplement: Supplementary file 1 [file ijms-25-03653-s001.zip › ijms-2850990-supplementary S2.pdf]

International Journal of
Molecular Sciences

MDPI

Supplementary File S2

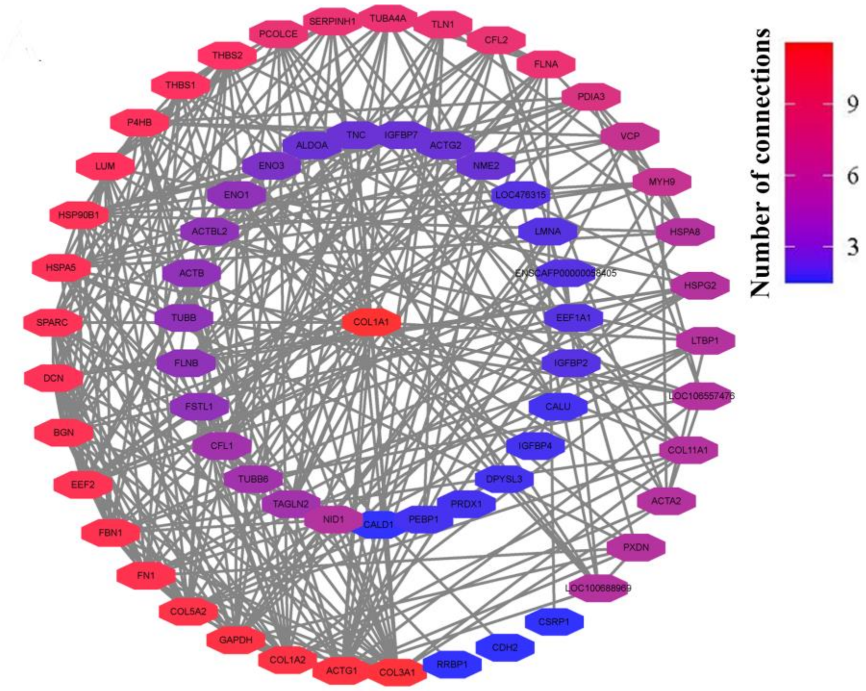

Figure S1. Protein-protein interaction (PPI) network of 66 most highly expressed proteins in MSC group. The color gradually changed from red to blue, indicating a decreasing degree of connectivity between proteins. The redder the color, the more connection number. The bluer the color, the less connection number (Figure 3B).

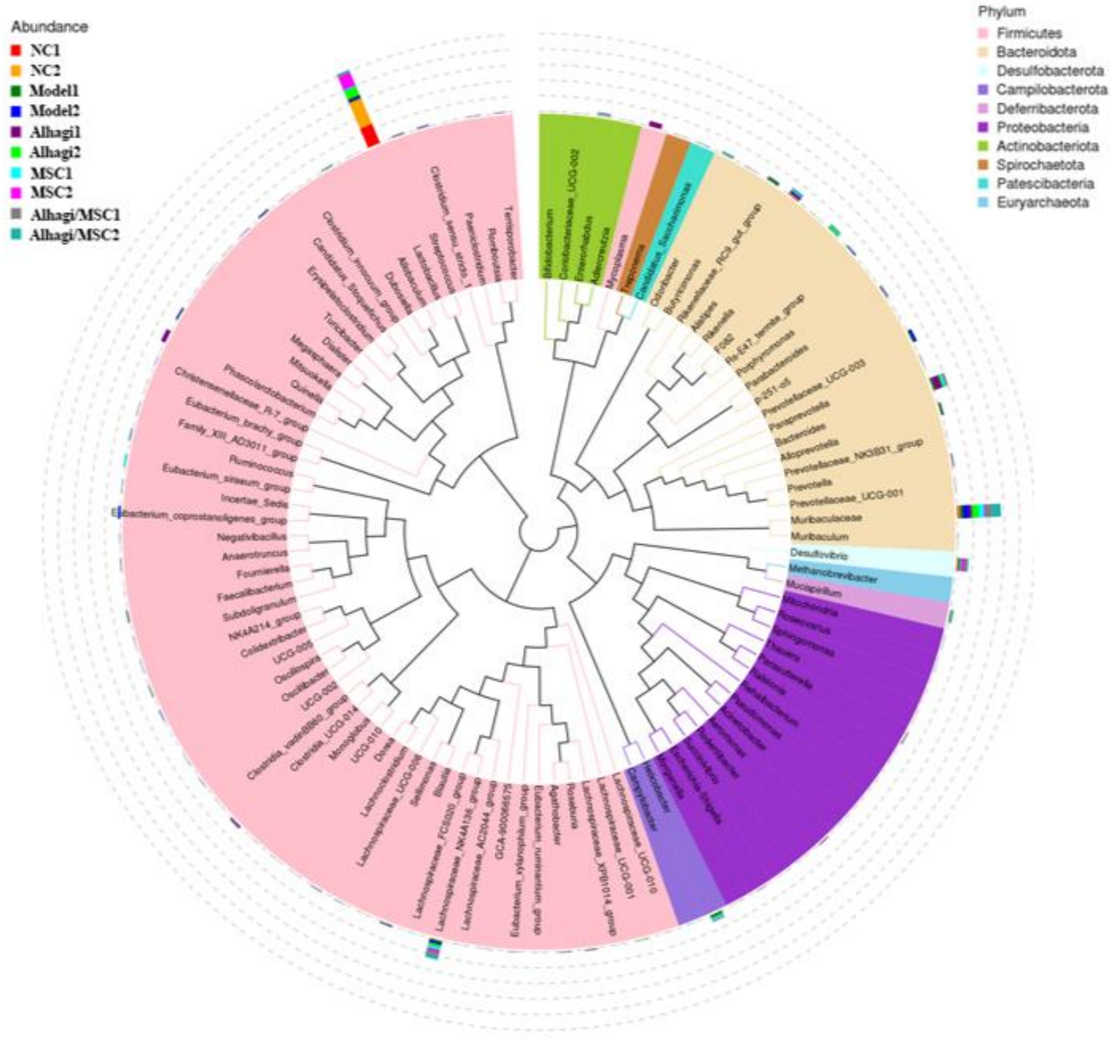

Figure S2. Phylogenetic tree of representative species sequences in genus level . The color of the sector indicates its corresponding phylum, and the inside of the sector is different genus within the same phylum. Stacked bar plots on the outside of the fan ring indicate the abundance of the genus in different group. The left legend is the grouping information, and the right legend is the phylum names (Figure 5E).
